# Aerobic training affects fatty acid composition of erythrocyte membranes

**DOI:** 10.1186/1476-511X-10-188

**Published:** 2011-10-22

**Authors:** Marina Marini, Provvidenza M Abruzzo, Alessandra Bolotta, Arsenio Veicsteinas, Carla Ferreri

**Affiliations:** 1 Department of Histology, Embryology, and Applied Biology, University of Bologna, Italy; 2 Department of Sport, Nutrition and Health Sciences, University of Milan, Italy; 3 Center for Sport Medicine, Don Gnocchi Foundation, Milan, Italy; 4 ISOF, CNR, Bologna, Italy

**Keywords:** aerobic training, lipidomics, erythrocyte membrane, trans fatty acids, rats

## Abstract

The effect of exercise training on the fatty acid composition of erythrocyte membranes was evaluated in an experimental animal model where rats were subjected to a ten-wk aerobic training. Five groups of rats were compared: sedentary rats at 19 or 23 wks of age, rats trained at moderate or high intensity sacrificed at 19 wks of age, and rats trained at high intensity, and sacrificed following 4 weeks of sedentary life. We had already demonstrated that cardioprotection correlates with training intensity and partially persists in detrained rats. Main findings are that rats trained at higher intensity display consistent signs of lipid peroxidation but a lower ω6/ω3 ratio and a lower content of trans fatty acids when compared to rats trained at lower intensity and to older sedentary rats. *Trans *fatty acids negatively affect cell membrane fluidity and permeability. Detrained rats showed intermediate values. Gene expression evaluation of selected enzymes involved in lipid biosynthesis revealed some of the adaptive mechanisms leading to the maintenance of membrane fatty acid homeostasis following exercise. The decrease in the amount of *trans *fatty and in the inflammatory pathways (i.e. ω6/ω3 ratio) in high-intensity trained rats underscores the protective effect of high intensity aerobic training.

## Introduction

Reactive Oxygen and Nitrogen Species (RONS) can be generated during physical activity by several different tissues, and increase cellular free radical content and circulating oxidative stress markers [1-3]. In particular, exhaustive endurance exercise generates excess RONS, leading to inflammatory responses and damage to DNA, proteins and lipids [4]. On the other hand, moderate exercise induces low levels of RONS, which are more involved in signaling pathways than in detrimental effects; in particular, adaptive responses may be generated, which ultimately counteract age-related degenerative diseases [1,5].

Membrane fatty acid composition is a very sensitive indicator of oxidative damage, since it may be affected by lipid peroxidation, which in turn may lead to the propagation of free radical reactions. Membrane fluidity and permeability is highly affected by fatty acid residues, namely saturated, monounsaturated and polyunsaturated fatty acids (SFA, MUFA and PUFA, respectively) and endogenous *trans *fatty acids (FA), and is strictly related to the ability to perform its many, various and demanding tasks. We asked how the exercise-related peroxidation of membrane FA reconciled with the well-known benefits of exercise training. Thus, we decided to investigate how a 10-wk aerobic training, which we previously showed to lead to adaptive responses, beneficial for the cardiocirculatory system [1,6-9], affected cell membrane FA composition. Gene expression evaluation of selected enzymes involved in lipid biosynthesis contributed to this lipidomic analysis, intended as an integrated approach to the study of the pathways ultimately leading to the membrane lipid composition. Erythrocyte membranes are thought to be a highly representative model for these studies [10].

## Materials and Methods

Rat care, exercise training and experimental groups were those described in a previous work [9]. Briefly, male Sprague-Dowley rats (2 months age) were trained at low (LT, ~60%VO_2max_), or high (HT, ~80%VO_2max_) intensity for 10 weeks on a rodent treadmill. A subgroup of HT rats was subjected to detraining for 4 weeks (HT-D). Two control groups were established: untrained rats sacrificed at the same age as LT and HT rats, i.e. at 19 weeks of age (UNT-Y), and untrained rats sacrificed 4 weeks later (UNT-O), in order to match the age of HT-D. Animal handling, training protocol and mode of sacrifice were approved by the Ethical Committee on the Use of Laboratory Animals (Milan, Protocol #3407 24/09/2007) according to the 86/609/CEE guidelines. Rats were fed standard diet at libitum; rat pellet was free of *trans *fatty acids, as controlled by lipid analysis [11].

Blood was drawn from deeply anesthetized animals by heart puncture, using Na-EDTA treated syringes. Liver was immediately removed, frozen in liquid nitrogen and stored at -80°C.

Lipidomic analysis was carried out as previously described [10]. Briefly, whole blood was centrifuged to remove plasma, then erythrocytes were lysed and membranes were separated by centrifugation. Lipids were extracted according to the method of Bligh and Dyer [12]. The phospholipid fraction was controlled by TLC as previously described [10], then treated with KOH/MeOH solution (0.5 M) for 10 min at room temperature and under stirring [13]. Fatty acid methyl esters (FAME) were extracted with n-hexane; the hexane phase was collected and dried with anhydrous Na_2_SO_4_. After filtration, the solvent was eliminated by evaporation at a rotating evaporator and the thin white film of the FAME was subsequently dissolved in a small volume of n-hexane. Approximately 1 μL of this solution was injected into the GC. A Varian CP-3800 gas chromatograph, with a flame ionization detector and an Rtx-2330 column (90% biscyanopropyl-10% phenylcyanopropyl polysiloxane capillary column; 60 m, 0.25 mm i.d., 0.20 μm film thickness) was used for the analysis. Temperature started from 165°C held for 3 min, followed by an increase of 1°C/min up to 195°C held for 40 min, followed by a second increase of 10°C/min up to 250°C held for 5 min. Helium was the carrier gas at the constant pressure of 29 psi.

Methyl esters were identified by comparison with the retention times of commercially available standards or trans fatty acid references obtained as described elsewhere [14]. By an external standard (C18:0) it was ascertained that a quantitative balance of fatty acids was present in each sample, so that the comparison of relative percentages reported in the Table is meaningful.

Quantitative gene expression of *Δ*-*9 Desaturase *(EC 1.14.19.1) and of *Elongases 1 *(EC 2.3.1.119) and *6 *(EC 2.3.1.16) was evaluated by qRT-PCR analysis on cDNA obtained from rat livers, according to the methods described in [8]. Primer sequences and amplicon lengths are reported in Table 1. For *Rattus norvegicus Δ*-*6 Desaturase *(EC 1.14.19.3) Gene Bank reports two sequences, a provisional one, Rn. 163176, and a predictive one, XM_002729187.1. When compared by BLAST 2Seq, no similarity was found between the sequences. Nevertheless, we carefully designed primers in order to amplify both of them. No amplification was detected with neither pair of primers by RealTime-qPCR in rat livers, thus showing that the information found in Gene Bank for *Rattus norvegicus Δ*-*6 Desaturase *sequence is inaccurate.

**Table 1:** Primer sequence and amplicon length of the genes studied with qRT-PCR

UniGene	Gene name (symbol)	Left primer sequence	Right primer sequence	Amplicon length (nt)
Rn 92211	*RPL13a*	GATGAACACCAACCCGTCTC	CACCATCCGCTTTTTCTTGT	175

Rn.1023	*Δ9*-*Desaturase *(EC 1.14.19.1)	CTGGTATCCTTGGGTGTGGA	ACATAGGGGTGGAGGTAGGG	100

Rn.29724	*Elongase 1 *(EC 2.3.1.119)	ATTCCTTCTCCCAGCCAGTT	GTGAGGAGAAAGGCCACAAG	162

Rn.46942	*Elongase 6 *(EC 2.3.1.16)	GCTCTGCATTGGGTAAAC	TAATCTACGCAGGCCCTTTG	162

The housekeeping gene *RPL13a *was used as reference gene for the relative expression calculations.

## Results and Discussion

**Figure 1:**
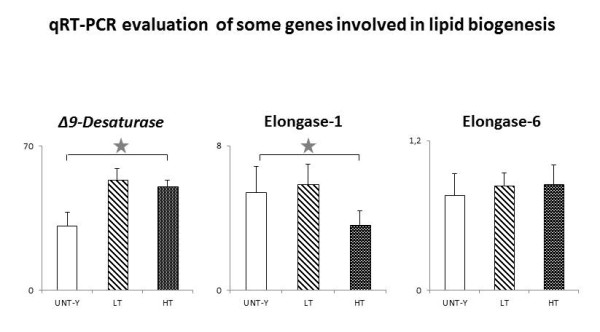
**qRT-PCR evaluation (mean ± SD) of some genes involved in lipid biogenesis expressed in rat livers**. Data were normalized to the housekeeping gene *RPL13a*. Star shows p ≤ 0.05 as evaluated by ANOVA analysis. Values are expressed in arbitrary units, setting to 100 the amount of *RPL13a *in 10 ng cDNA input.

Table 2 shows the lipidomic profile of the erythrocyte membranes from the five experimental groups of rats and Figure 1 shows the mRNA abundance of three relevant enzymes involved in lipid biosynthesis, evaluated only in control and exercised rats. Comparisons among the groups suggest the following considerations about the effects of exercise training: i) a decrease in arachidonic acid, an increase in SFA and SFA/MUFA and a corresponding decrease in PUFA, Unsaturation Index (U.I.) and Peroxidation Index (P.I.) all indicate that exercise training is associated with an increased PUFA mobilization from membranes, probably due to increased generation of RONS [2,9] and to peroxidation processes; the exercise-related decrease in arachidonic acid has been already reported by others [15] to occur in muscle membranes; ii) the presence of trans FA, especially the arachidonic acid isomers, which are absent in UNT and are strongly increased in LT rats, may be ascribed to radical stress and suggests that HT rats may develop a better control of radical stress than LT rats; studying cardiomyocytes, we recently demonstrated that HT rats develop higher levels of HSP70i protein and SOD activity than LT rats [9]; iii) a decrease of ω6/ω3 in HT rats points to an exercise-related reduction of inflammatory responses; iv) an increase in stearic acid amount in HT rats is related to the mobilization of PUFA from membranes and the subsequent insertion of SFA for membrane lipid regeneration; the consequent increase in membrane stiffness may be a toll to pay in exchange of increased membrane stability and decreased P.I., both valuable during training, but requiring v) the upregulation of *Δ*-*9 Desaturase *gene expression (Figure 1) in order to restore PUFA content and membrane fluidity; as a side note, we acknowledge that others have recently described the occurrence of training-related downregulation of *Δ*-*9 Desaturase *[16]; in the absence of information about membrane lipid composition in the experimental system reported in [16], such discrepancy cannot be explained, however one should remember that *Δ*-*9 Desaturase *is also involved in the de novo synthesis of storage FA [17]; vi) an increase in linoleic acid amount in trained rats suggests that *Δ*-*6 Desaturase *might be downregulated, an information which we had been unable to gather (see above in the Methods section); since trained animals tended to eat less than the untrained ones (Veicsteinas, personal communication), the increase in linoleic acid is not related to their feeding; vii) a decrease in vaccenic acid is probably due to the decrease of *Elongase 1 *gene expression (Figure 1); the adaptive meaning for this last change is difficult to understand.

**Table 2:** Individual fatty acids in erythrocyte membranes were expressed as relative percentages of the total fatty acids identified

FAME	UNT-Y	LT	HT	HT-D	UNT-O
	N = 8	N = 8	N = 7	N = 4	N = 4
Palmitic, 16:0	36.2 ± 2.9	37.0 ± 2.4	35.2 ±1.3^d^	37.3 ± 1.5^a,e^	38.0 ± 1.7^a^

Palmitoleic, 16:1	0.3 ± 0.2	0.4 ± 0.05	0.3 ± 0.1^d^	0.4 ± 0.0^a,d^	0.5 ± 0.1^a^

Stearic, 18:0	16.8 ± 0.2	15.0 ± 0.9	18.3 ± 1.4^c^	19.4 ± 1.5^a,c,g^	14.2 ± 0.8^b^

*trans *18:1	0.1 ± 0.1	0.2 ± 0.1	0.2 ± 0.2	0.1 ± 0.0	0.1 ± 0.0

Oleic, 9*c*-18:1	6.2 ± 0.3	6.5 ± 0.8	6.0 ± 0.6	5.4 ± 0.3^b,h^	5.9 ± 0.8

Vaccenic, 11*c*-18:1	3.3 ± 0.2	3.4 ± 0.3^e^	2.7 ± 0.2^b,d^	2.9 ± 0.3^d^	3.4 ± 0.4

Linoleic, 9*c*,12*c*-18:2	10.8 ± 0.5	13.2 ± 0.6	12.7 ± 0.8^a^	10.5 ± 1.3 ^d,f,h^	13.5 ± 0.8^a^

DGLA, 20:3 n-6	0.2 ± 0.2	0.2 ± 0.1	0.4 ± 0.2	0.4 ± 0.1^g^	0.4 ± 0.2

Arachidonic, 20:4 n-6	26.6 ± 1.2	19.9 ± 1.5	20.7 ± 0.9^b^	19.3 ± 1.0^b,f^	20.0 ± 1.4^b^

*trans-20:4*	0.0 ± 0.0	1.8 ± 0.3^e^	0.2 ± 0.1^a,d^	0.6 ± 0.2^a,d,e,h^	1.6 ± 0.6^a^

EPA n-3	0.4 ± 0.3	0.2 ± 0.0	0.4 ± 0.4	0.3 ± 0.1^g^	0.4 ± 0.3

DHA n-3	2.0 ± 0.3	1.6 ± 0.1^f^	2.4 ± 0.3^c^	2.65 ± 0.3^a,c,h^	1.8 ± 0.2

Total *trans*	0.1 ± 0.1	1.9 ± 0.3^e^	0.4 ± 0.2^d^	0.7 ± 0.2^a,d,e,h^	1.7 ± 0.6^a^

SFA	49.9 ± 1.2	52.0 ± 2.5	53.5 ± 1.7^a^	57.2 ± 2.12^a,c,e,g^	52.2 ± 2.0

MUFA	9.6 ± 0.0	10.3 ± 0.8^e^	9.0 ± 0.8^d^	8.7 ± 0.4^b,d,h^	9.9 ± 0.7

PUFA	40.1 ± 1.3	35.0 ± 1.7	36.6 ± 1.5^b^	33.2 ± 1.9^b,d,f^	36.0 ± 1.3^b^

SFA/MUFA	5.2 ± 0.1	5.1 ± 0.5	6.0 ± 0.7^a^,^c^	6.6 ± 0.4^a,c,g^	5.3 ± 0.6

ω6/ω3	15.8 ± 2.9	18.8 ± 0.8^e^	12.1 ± 1.6^b,d^	10.5 ± 1.3^b,d,h^	15.9 ± 3.0

U.I.	152.8 ± 6.1	127.1 ± 6.4	134.9 ± 5.5^b^	125.51 ± 6.2^b,f^	130.8 ± 7.3^b^

PI.	136.8 ± 7.3	107.0 ± 6.9^e^	118.3 ± 4.7,^b,c^	111.6 ± 6.1^b,f^	111.4 ± 8.1^b^

All data are presented as means ± SD and are expressed as % of peak areas in the gas chromatograms. The identification of the peaks have been performed by authentic references and the identified peaks were >97% of the total GC peaks.

Superscripts show the results of ANOVA analysis, as follows:

^a^Higher than UNT-Y, p < 0.05;

^b^Lower than UNT-Y, p < 0.05;

^c^Higher than UNT-O, p < 0.05;

^d^Lower than UNT-O, p < 0.05;

^e^Higher than HT, p < 0.05;

^f^Lower than HT, p < 0.05;

^g^Higher than LT, p < 0.05;

^h^Lower than LT, p < 0.05.

Changes of the lipidomic profile of HT-D rats are better understood in comparison with the profile of the UNT-O group, since the effect of age should be considered. In fact, older rats, compared to younger ones, display the same tendency to increased SFA and decreased PUFA, U.I. and P.I. observed in HT rats, as a possible result of lipid peroxidation reactions. This does not come unexpected if interpreted in the light of the free radical theory of aging [18], one of the most accredited theories on aging. HT-D rats, however, differ from UNT-O for some parameters, and notably in the trans fatty acid content, which is intermediate between that of HT (lower) and that of UNT-O (higher), as if some of the endogenous defenses built up during training continued to protect membrane lipids from radical attack during the 4 wks of detraining.

Increasing epidemiologic and biochemical evidence suggests that excessive *trans *FA significantly increase the risk for cardiovascular events [19]. Apart from the dietary contribution, the conversion of lipids from *cis *to *trans *geometry involves the thiyl radical catalyzed isomerization [20], a process that may occur under radical stress conditions [11]. Thus, the fact that high intensity training apparently induces protection against *trans *fat isomerization underlines one of the many beneficial effects of aerobic exercise.

## Abbreviations

FA: Fatty Acid; FAME: Fatty Acid Methyl Esters; DGLA: Dihomo-Gamma-Linolenic Acid; EPA: eicosapentaenoic acid; DHA: docoesaenoic acid, SFA: Saturated Fatty Acids; MUFA: Monounsaturated Fatty Acids; PUFA: Polyunsaturated Fatty Acids; U.I., Unsaturation Index; P.I.: Peroxidation Index.

## Competing interests

The authors declare that they have no competing interests.

## Authors' contributions

MM conceived of the study, supervised the gene expression study and drafted the manuscript. PMA carried out the gene expression study and helped to draft the manuscript. AB participated in the gene expression study and coordinated the experimental work. AS participated in the design of the study and supervised the animal training. CF participated in the design of the study, performed the lipidomic analysis and helped to draft the manuscript. All authors read and approved the final manuscript.

## Authors' information

MM is associate professor of cell biology and genetics. PMA is a post-doc and AB is a PhD student. MM, PMA and AB share a common interest in the role of ROS/RNS and oxidative/radical stress in health and disease. AS is an MD, full professor of physiology with a long-standing interest in exercise. CF is Senior Researcher in National Council of Research and heads the Lipid Units of her Institute.
